# 1-(2,4-Di­nitro­phen­yl)-2-[(*E*)-(3,4,5-tri­meth­oxy­benzyl­idene)]hydrazine

**DOI:** 10.1107/S1600536814001238

**Published:** 2014-01-22

**Authors:** Suchada Chantrapromma, Pumsak Ruanwas, Nawong Boonnak, C. S. Chidan Kumar, Hoong-Kun Fun

**Affiliations:** a Department of Chemistry, Faculty of Science, Prince of Songkla University, Hat-Yai, Songkhla 90112, Thailand; b Faculty of Traditional Thai Medicine, Prince of Songkla University, Hat-Yai, Songkhla 90112, Thailand; c X-ray Crystallography Unit, School of Physics, Universiti Sains Malaysia, 11800 USM, Penang, Malaysia

## Abstract

Mol­ecules of the title compound, C_16_H_16_N_4_O_7_, are not planar with a dihedral angle of 5.50 (11)° between the substituted benzene rings. The two *meta*-meth­oxy groups of the 3,4,5-tri­meth­oxy­benzene moiety lie in the plane of the attached ring [C_meth­yl_–O–C–C torsion angles −0.1 (4)° and −3.7 (3)°] while the *para*-meth­oxy substituent lies out of the plane [C_meth­yl_—O—C—C, −86.0 (3)°]. An intra­molecular N—H⋯O hydrogen bond involving the 2-nitro substituent generates an *S*(6) ring motif. In the crystal structure, mol­ecules are linked by weak C—H⋯O inter­actions into screw chains, that are arranged into a sheet parallel to the *bc* plane. These sheets are connected by π–π stacking inter­actions between the nitro and meth­oxy substituted aromatic rings with a centroid–centroid separation of 3.9420 (13) Å. C—H⋯π contacts further stabilize the two-dimensional network.

## Related literature   

For background to the biological activity of hydro­zones, see: Angelusiu *et al.* (2010 ▶); Cui *et al.* (2010 ▶), Gokce *et al.* (2009 ▶); Molyneux (2004 ▶); Sathyadevi *et al.* (2012 ▶); Wang *et al.* (2009 ▶). For details of hydrogen-bond motifs, see: Bernstein *et al.* (1995 ▶). For related structures, see: Fun *et al.* (2011 ▶, 2012 ▶, 2013 ▶).
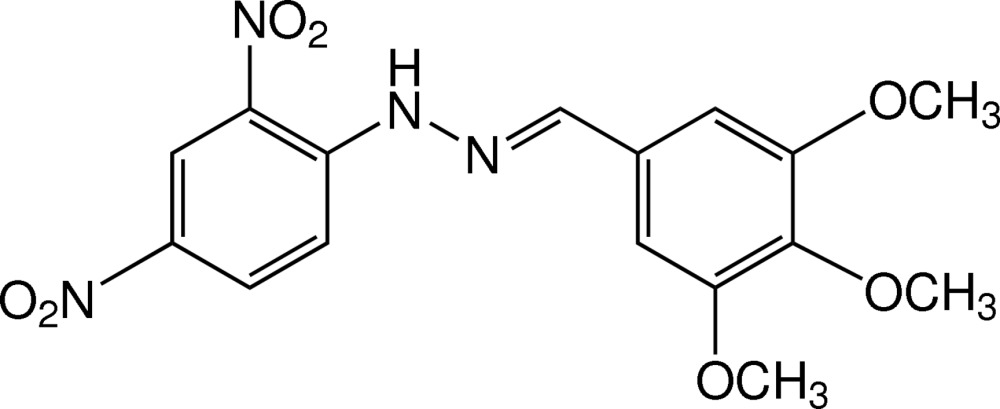


## Experimental   

### 

#### Crystal data  

C_16_H_16_N_4_O_7_
*M* *_r_* = 376.33
Orthorhombic, 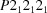

*a* = 7.4724 (4) Å
*b* = 14.3106 (7) Å
*c* = 16.1549 (7) Å
*V* = 1727.52 (15) Å^3^
*Z* = 4
Mo *K*α radiation
μ = 0.12 mm^−1^
*T* = 100 K
0.58 × 0.30 × 0.24 mm

#### Data collection  

Bruker APEXII CCD area detector diffractometer
Absorption correction: multi-scan (*SADABS*; Bruker, 2009 ▶) *T*_min_ = 0.936, *T*_max_ = 0.972
20053 measured reflections
2855 independent reflections
2243 reflections with *I* > 2σ(*I*)
*R*_int_ = 0.039

#### Refinement  

*R*[*F* ^2^ > 2σ(*F* ^2^)] = 0.048
*wR*(*F* ^2^) = 0.107
*S* = 1.08
2855 reflections
251 parameters
H atoms treated by a mixture of independent and constrained refinement
Δρ_max_ = 0.14 e Å^−3^
Δρ_min_ = −0.22 e Å^−3^

### 

Data collection: *APEX2* (Bruker, 2009 ▶); cell refinement: *SAINT* (Bruker, 2009 ▶); data reduction: *SAINT*; program(s) used to solve structure: *SHELXTL* (Sheldrick, 2008 ▶); program(s) used to refine structure: *SHELXTL*; molecular graphics: *SHELXTL*; software used to prepare material for publication: *SHELXTL*, *PLATON* (Spek, 2009 ▶), *Mercury* (Macrae *et al.*, 2008 ▶) and *publCIF* (Westrip, 2010 ▶).

## Supplementary Material

Crystal structure: contains datablock(s) global, I. DOI: 10.1107/S1600536814001238/sj5380sup1.cif

Structure factors: contains datablock(s) I. DOI: 10.1107/S1600536814001238/sj5380Isup2.hkl

CCDC reference:

Additional supporting information:  crystallographic information; 3D view; checkCIF report

## Figures and Tables

**Table 1:** Hydrogen-bond geometry (Å, °) *Cg*1 is the centroid of the C1–C6 benzene ring.

*D*—H⋯*A*	*D*—H	H⋯*A*	*D*⋯*A*	*D*—H⋯*A*
N1—H1*N*1⋯O1	0.82 (2)	2.04 (2)	2.624 (3)	129 (2)
C16—H16*C*⋯O2^i^	0.96	2.44	3.169 (4)	133
C14—H14*B*⋯*Cg*1^ii^	0.96	2.89	3.514 (3)	123

Symmetry codes: (i) 
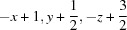
; (ii) 
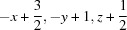
.

**Fig. 1.**
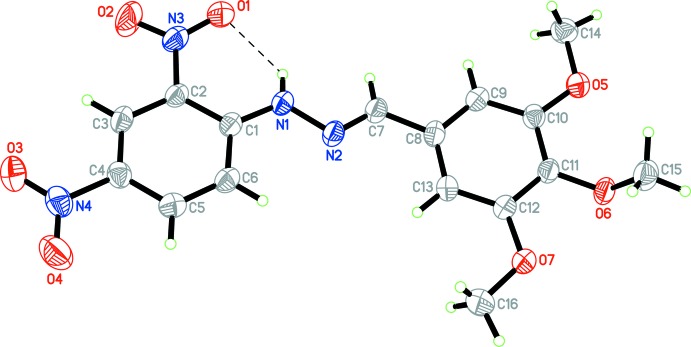
The molecular structure of (I), showing 40% probability displacement ellipsoids and the atom-numbering scheme. An intramolecular N—H···O hydrogen bond is shown as a dashed line.

**Fig. 2.**
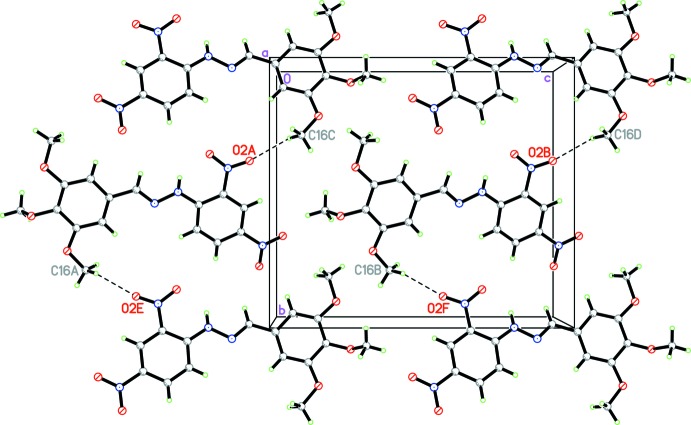
The crystal packing of (I) viewed along the a axis. Hydrogen bonds are shown as dashed lines.

**Fig. 3.**
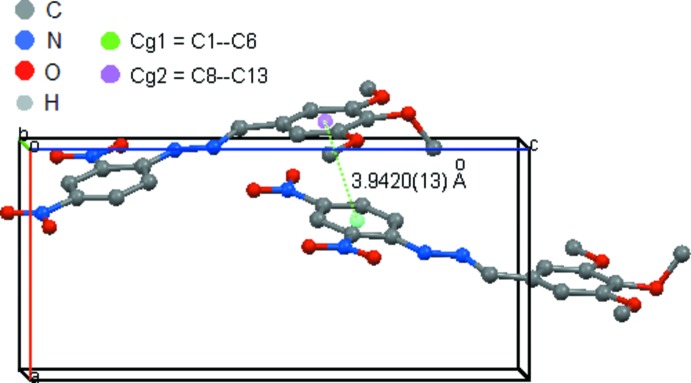
The π···π stacking interaction between the two substituted benzene rings. H atoms were omitted for clarity.

## supplementary crystallographic information

### 1. Comment

There are numerous reports of the various biological activities of hydrazones and their complexes which show antibacterial, antifungal, antitumor, anti-inflammatory and antioxidant activities (Angelusiu *et al.*, 2010; Cui *et al.*, 2010; Gokce *et al.*, 2009; Sathyadevi *et al.*, 2012 and Wang *et al.*, 2009).

In previous works, we synthesized a number of hydrazone derivatives from the reaction of 2,4-dinitrophenylhydrazine and various substituted aldehydes (Fun *et al.*, 2011, 2012 and 2013). The title hydrazone (I) was again synthesized using 2,4-dinitrophenylhydrazine but with 3,4,5-trimethoxybenzaldehyde as the aldehyde. Our evaluation of the antioxidant activity of (I) by the DPPH free radical scavenging method [DPPH = 2,2-diphenyl-1-picrylhydrazyl] (Molyneux, 2004) showed that it displays weak antioxidant activity with 17.6% inhibition. This further confirms observations from previous works (Fun *et al.*, 2011, 2012 and 2013) that the antioxidant ability of such compounds is controlled by the number and substitution pattern of the methoxy substituents. Herein we report the synthesis and crystal structure of (I).

In Fig. 1, the whole molecular structure of (I), C_16_H_16_N_4_O_7_ is not planar, with the dihedral angle between the two substituted benzene rings being 5.50 (11)°. Both nitro groups lie close to the plane of the attached benzene ring [torsion angles O1–N3–C2–C1 = 4.7 (4)°, O2–N3–C2–C3 = 5.1 (4)°, O3–N4–C4–C3 = -1.9 (3)° and O4–N4–C4–C5 = -0.8 (3)°]. The mean plane through the central N1/N2/C7 bridge makes dihedral angles of 6.8 (2)° and 12.3 (2)° with the two adjacent C1–C6 and C8–C13 benzene rings, respectively. The three methoxy groups of the 3,4,5-trimethoxyphenyl unit have two different orientations: the two *meta*-methoxy groups (at C10 and C12) are co-planar with the benzene ring plane (Fig. 3) with torsion angles C14–O5–C10–C9 = -3.7 (3)° and C16–O7–C12–C13 = -0.1 (4)° whereas the *para*-methoxy substituent (at C11) is out of the plane with the torsion angle C15–O6–C11–C10 = -86.0 (3)°. An intramolecular N1—H1N1···O1 hydrogen bond (Fig. 1 and Table 1) generates an S(6) ring motif (Bernstein *et al.*, 1995). Bond distances for (I) are comparable to those found in closely related structures (Fun *et al.*, 2011, 2012, 2013).

In the crystal packing (Fig. 2), molecules are linked by a weak intermolecular C16—H16C···O2 interaction (Table 1) into screw chains. These chains are arranged into sheets and further stacked along the *a* axis by π···π interactions with distances of Cg_1_···Cg_2_^iii, iv^ = 3.9420 (13) Å [Cg1 and Cg2 are the centroids of the C1···C6 and C8···C13 benzene rings, respectively; symmetry codes (iii) = 1/2-x, 1-y, -1/2+z and (iv) = 1/2+x, 1-y, 1/2+z] (Fig. 3). The molecules are linked into two dimensional network by weak C—H···O interactions. These C—H···π contacts further stabilize the two-dimensional network (Table 1).

### 2. Experimental

The title compound (I) was synthesized by dissolving 2,4-dinitrophenylhydrazine (0.40 g, 2 mmol) in ethanol (10.00 ml) and H_2_SO_4_ (conc.) (98 %, 0.50 ml) was added slowly with stirring. A solution of 3,4,5-trimethoxybenzaldehyde (0.40 g, 2 mmol) in ethanol (20.00 ml) was then added to the solution with continuous stirring for 1 hr, yielding an orange solid which was filtered off and washed with methanol. Orange block-shaped single crystals of the title compound suitable for *X*-ray structure determination were recrystallized from acetone by slow evaporation of the solvent at room temperature over a few weeks, Mp. 496–497 K.

### 3. Refinement

The hydrazine H atom was located from a difference Fourier map and refined freely. The remaining H atoms were positioned geometrically and allowed to ride on their parent atoms, with d(C-H) = 0.93 Å for CH and aromatic, and 0.96 Å for CH_3_ atoms. The *U*_iso_ values were constrained to be 1.5*U*_eq_ of the carrier atom for methyl H atoms and 1.2*U*_eq_ for the remaining H atoms. A rotating group model was used for the methyl groups.

### Crystal data

**Table:**

C_16_H_16_N_4_O_7_	*D*_x_ = 1.447 Mg m^−^^3^
*M**_r_* = 376.33	Melting point = 496–497 K
Orthorhombic, *P*2_1_2_1_2_1_	Mo *K*α radiation, λ = 0.71073 Å
Hall symbol: P 2ac 2ab	Cell parameters from 2855 reflections
*a* = 7.4724 (4) Å	θ = 2.5–30.0°
*b* = 14.3106 (7) Å	µ = 0.12 mm^−^^1^
*c* = 16.1549 (7) Å	*T* = 100 K
*V* = 1727.52 (15) Å^3^	Block, orange
*Z* = 4	0.58 × 0.30 × 0.24 mm
*F*(000) = 784	

### Data collection

**Table:**

Bruker APEXII CCD area detector diffractometer	2855 independent reflections
Radiation source: sealed tube	2243 reflections with *I* > 2σ(*I*)
Graphite monochromator	*R*_int_ = 0.039
φ and ω scans	θ_max_ = 30.0°, θ_min_ = 2.5°
Absorption correction: multi-scan (*SADABS*; Bruker, 2009)	*h* = −10→10
*T*_min_ = 0.936, *T*_max_ = 0.972	*k* = −17→20
20053 measured reflections	*l* = −22→22

### Refinement

**Table:**

Refinement on *F*^2^	Primary atom site location: structure-invariant direct methods
Least-squares matrix: full	Secondary atom site location: difference Fourier map
*R*[*F*^2^ > 2σ(*F*^2^)] = 0.048	Hydrogen site location: inferred from neighbouring sites
*wR*(*F*^2^) = 0.107	H atoms treated by a mixture of independent and constrained refinement
*S* = 1.08	*w* = 1/[σ^2^(*F*_o_^2^) + (0.0489*P*)^2^ + 0.1811*P*] where *P* = (*F*_o_^2^ + 2*F*_c_^2^)/3
2855 reflections	(Δ/σ)_max_ = 0.001
251 parameters	Δρ_max_ = 0.14 e Å^−^^3^
0 restraints	Δρ_min_ = −0.22 e Å^−^^3^

### Special details

**Table:**

Experimental. The crystal was placed in the cold stream of an Oxford Cryosystems Cobra open-flow nitrogen cryostat (Cosier & Glazer, 1986) operating at 120.0 (1) K.
Geometry. All esds (except the esd in the dihedral angle between two l.s. planes) are estimated using the full covariance matrix. The cell esds are taken into account individually in the estimation of esds in distances, angles and torsion angles; correlations between esds in cell parameters are only used when they are defined by crystal symmetry. An approximate (isotropic) treatment of cell esds is used for estimating esds involving l.s. planes.
Refinement. Refinement of F^2^ against ALL reflections. The weighted R-factor wR and goodness of fit S are based on F^2^, conventional R-factors R are based on F, with F set to zero for negative F^2^. The threshold expression of F^2^ > 2sigma(F^2^) is used only for calculating R-factors(gt) etc. and is not relevant to the choice of reflections for refinement. R-factors based on F^2^ are statistically about twice as large as those based on F, and R- factors based on ALL data will be even larger.

### Fractional atomic coordinates and isotropic or equivalent isotropic displacement parameters (Å^2^)

**Table:**

	*x*	*y*	*z*	*U*_iso_*/*U*_eq_	
O1	0.4866 (3)	0.35711 (12)	0.69622 (11)	0.0629 (5)	
O2	0.4403 (4)	0.38109 (15)	0.56725 (12)	0.0928 (9)	
O3	0.2099 (3)	0.66252 (15)	0.46266 (11)	0.0705 (6)	
O4	0.1541 (4)	0.77886 (16)	0.54355 (13)	0.0804 (7)	
O5	0.6955 (2)	0.40878 (11)	1.21814 (9)	0.0482 (4)	
O6	0.6333 (2)	0.58591 (11)	1.26663 (9)	0.0467 (4)	
O7	0.5222 (3)	0.71519 (11)	1.16062 (10)	0.0620 (5)	
N1	0.4733 (3)	0.49707 (14)	0.80101 (11)	0.0419 (4)	
H1N1	0.520 (3)	0.4462 (17)	0.7932 (14)	0.041 (7)*	
N2	0.4873 (3)	0.53900 (13)	0.87742 (10)	0.0436 (4)	
N3	0.4436 (3)	0.40829 (14)	0.63849 (13)	0.0516 (5)	
N4	0.2094 (3)	0.69931 (16)	0.53100 (13)	0.0535 (5)	
C1	0.4052 (3)	0.54403 (15)	0.73618 (12)	0.0374 (4)	
C2	0.3933 (3)	0.50496 (15)	0.65539 (13)	0.0391 (5)	
C3	0.3317 (3)	0.55573 (17)	0.58869 (13)	0.0428 (5)	
H3A	0.3279	0.5292	0.5362	0.051*	
C4	0.2756 (3)	0.64651 (16)	0.60122 (14)	0.0425 (5)	
C5	0.2773 (3)	0.68693 (16)	0.67997 (14)	0.0445 (5)	
H5A	0.2349	0.7474	0.6878	0.053*	
C6	0.3416 (3)	0.63669 (16)	0.74534 (14)	0.0434 (5)	
H6A	0.3438	0.6642	0.7975	0.052*	
C7	0.5672 (3)	0.48885 (17)	0.93111 (13)	0.0439 (5)	
H7A	0.6149	0.4317	0.9148	0.053*	
C8	0.5867 (3)	0.51809 (16)	1.01780 (13)	0.0422 (5)	
C9	0.6408 (3)	0.45032 (17)	1.07387 (13)	0.0419 (5)	
H9A	0.6717	0.3909	1.0554	0.050*	
C10	0.6487 (3)	0.47149 (16)	1.15789 (13)	0.0401 (5)	
C11	0.6106 (3)	0.56190 (16)	1.18476 (13)	0.0408 (5)	
C12	0.5579 (3)	0.63001 (15)	1.12739 (14)	0.0446 (5)	
C13	0.5446 (4)	0.60796 (16)	1.04410 (13)	0.0467 (6)	
H13A	0.5080	0.6529	1.0061	0.056*	
C14	0.7279 (4)	0.31452 (17)	1.19216 (17)	0.0543 (6)	
H14A	0.7677	0.2782	1.2386	0.081*	
H14B	0.8182	0.3140	1.1499	0.081*	
H14C	0.6193	0.2882	1.1706	0.081*	
C15	0.4822 (4)	0.5655 (2)	1.31779 (15)	0.0589 (7)	
H15A	0.5073	0.5840	1.3737	0.088*	
H15B	0.4581	0.4996	1.3162	0.088*	
H15C	0.3797	0.5991	1.2978	0.088*	
C16	0.4671 (7)	0.78726 (19)	1.10517 (18)	0.0905 (13)	
H16A	0.4494	0.8442	1.1355	0.136*	
H16B	0.3571	0.7695	1.0788	0.136*	
H16C	0.5578	0.7966	1.0639	0.136*	

### Atomic displacement parameters (Å^2^)

**Table:**

	*U*^11^	*U*^22^	*U*^33^	*U*^12^	*U*^13^	*U*^23^
O1	0.0928 (15)	0.0415 (8)	0.0545 (10)	0.0056 (11)	−0.0095 (11)	−0.0040 (8)
O2	0.157 (3)	0.0725 (13)	0.0492 (11)	0.0356 (16)	−0.0179 (14)	−0.0286 (10)
O3	0.0917 (15)	0.0789 (13)	0.0410 (10)	−0.0034 (13)	−0.0048 (10)	0.0092 (9)
O4	0.1078 (19)	0.0642 (13)	0.0691 (13)	0.0249 (14)	0.0022 (13)	0.0156 (10)
O5	0.0592 (11)	0.0459 (9)	0.0394 (8)	0.0015 (8)	−0.0075 (7)	0.0025 (7)
O6	0.0545 (10)	0.0552 (9)	0.0304 (7)	−0.0098 (8)	−0.0006 (7)	−0.0059 (7)
O7	0.1072 (16)	0.0410 (8)	0.0376 (8)	0.0064 (11)	0.0011 (10)	−0.0034 (7)
N1	0.0522 (12)	0.0412 (10)	0.0322 (9)	0.0014 (10)	0.0006 (8)	−0.0061 (7)
N2	0.0522 (12)	0.0476 (10)	0.0309 (8)	−0.0034 (9)	0.0013 (8)	−0.0056 (7)
N3	0.0627 (14)	0.0454 (10)	0.0468 (11)	0.0015 (11)	−0.0025 (10)	−0.0120 (9)
N4	0.0556 (13)	0.0585 (13)	0.0465 (12)	−0.0041 (11)	0.0039 (10)	0.0121 (10)
C1	0.0364 (11)	0.0419 (11)	0.0338 (10)	−0.0058 (9)	0.0027 (8)	−0.0034 (8)
C2	0.0432 (12)	0.0397 (11)	0.0345 (10)	−0.0020 (10)	0.0015 (9)	−0.0079 (9)
C3	0.0442 (12)	0.0530 (13)	0.0313 (10)	−0.0056 (11)	0.0008 (9)	−0.0057 (9)
C4	0.0409 (12)	0.0473 (13)	0.0393 (11)	−0.0054 (10)	0.0002 (9)	0.0051 (9)
C5	0.0461 (12)	0.0395 (12)	0.0479 (12)	−0.0003 (10)	0.0027 (11)	−0.0031 (10)
C6	0.0472 (13)	0.0444 (12)	0.0387 (11)	−0.0001 (11)	0.0033 (10)	−0.0086 (9)
C7	0.0505 (14)	0.0474 (12)	0.0338 (10)	−0.0023 (11)	0.0026 (10)	−0.0050 (9)
C8	0.0451 (13)	0.0502 (13)	0.0312 (10)	−0.0030 (10)	−0.0011 (9)	−0.0019 (8)
C9	0.0438 (13)	0.0434 (12)	0.0384 (11)	−0.0010 (10)	−0.0004 (10)	−0.0060 (9)
C10	0.0389 (12)	0.0452 (12)	0.0361 (10)	−0.0036 (10)	−0.0039 (9)	0.0004 (8)
C11	0.0455 (12)	0.0455 (12)	0.0315 (10)	−0.0065 (10)	−0.0008 (9)	−0.0027 (9)
C12	0.0573 (15)	0.0409 (11)	0.0356 (10)	−0.0028 (11)	0.0012 (10)	−0.0036 (9)
C13	0.0617 (16)	0.0462 (12)	0.0321 (10)	−0.0020 (12)	0.0023 (10)	0.0012 (8)
C14	0.0585 (15)	0.0468 (14)	0.0576 (15)	0.0035 (12)	−0.0082 (13)	0.0014 (11)
C15	0.0664 (17)	0.0673 (16)	0.0429 (13)	−0.0129 (15)	0.0121 (13)	−0.0077 (11)
C16	0.172 (4)	0.0442 (14)	0.0557 (16)	0.019 (2)	−0.002 (2)	0.0042 (12)

### Geometric parameters (Å, º)

**Table:**

O1—N3	1.229 (3)	C5—C6	1.365 (3)
O2—N3	1.215 (3)	C5—H5A	0.9300
O3—N4	1.223 (3)	C6—H6A	0.9300
O4—N4	1.228 (3)	C7—C8	1.469 (3)
O5—C10	1.369 (3)	C7—H7A	0.9300
O5—C14	1.433 (3)	C8—C9	1.387 (3)
O6—C11	1.377 (2)	C8—C13	1.391 (3)
O6—C15	1.430 (3)	C9—C10	1.392 (3)
O7—C12	1.358 (3)	C9—H9A	0.9300
O7—C16	1.427 (3)	C10—C11	1.394 (3)
N1—C1	1.344 (3)	C11—C12	1.402 (3)
N1—N2	1.376 (2)	C12—C13	1.386 (3)
N1—H1N1	0.82 (2)	C13—H13A	0.9300
N2—C7	1.274 (3)	C14—H14A	0.9600
N3—C2	1.459 (3)	C14—H14B	0.9600
N4—C4	1.450 (3)	C14—H14C	0.9600
C1—C6	1.416 (3)	C15—H15A	0.9600
C1—C2	1.423 (3)	C15—H15B	0.9600
C2—C3	1.379 (3)	C15—H15C	0.9600
C3—C4	1.380 (3)	C16—H16A	0.9600
C3—H3A	0.9300	C16—H16B	0.9600
C4—C5	1.398 (3)	C16—H16C	0.9600
			
C10—O5—C14	116.87 (18)	C9—C8—C7	116.9 (2)
C11—O6—C15	114.02 (18)	C13—C8—C7	122.2 (2)
C12—O7—C16	117.21 (18)	C8—C9—C10	119.8 (2)
C1—N1—N2	120.63 (18)	C8—C9—H9A	120.1
C1—N1—H1N1	119.1 (17)	C10—C9—H9A	120.1
N2—N1—H1N1	119.6 (17)	O5—C10—C9	124.1 (2)
C7—N2—N1	113.60 (19)	O5—C10—C11	116.05 (19)
O2—N3—O1	122.2 (2)	C9—C10—C11	119.8 (2)
O2—N3—C2	118.4 (2)	O6—C11—C10	120.4 (2)
O1—N3—C2	119.38 (19)	O6—C11—C12	119.7 (2)
O3—N4—O4	123.3 (2)	C10—C11—C12	119.80 (19)
O3—N4—C4	118.7 (2)	O7—C12—C13	125.0 (2)
O4—N4—C4	118.0 (2)	O7—C12—C11	114.71 (18)
N1—C1—C6	120.90 (19)	C13—C12—C11	120.3 (2)
N1—C1—C2	122.81 (19)	C12—C13—C8	119.4 (2)
C6—C1—C2	116.3 (2)	C12—C13—H13A	120.3
C3—C2—C1	122.1 (2)	C8—C13—H13A	120.3
C3—C2—N3	116.06 (18)	O5—C14—H14A	109.5
C1—C2—N3	121.9 (2)	O5—C14—H14B	109.5
C2—C3—C4	118.87 (19)	H14A—C14—H14B	109.5
C2—C3—H3A	120.6	O5—C14—H14C	109.5
C4—C3—H3A	120.6	H14A—C14—H14C	109.5
C3—C4—C5	121.4 (2)	H14B—C14—H14C	109.5
C3—C4—N4	118.6 (2)	O6—C15—H15A	109.5
C5—C4—N4	120.0 (2)	O6—C15—H15B	109.5
C6—C5—C4	119.3 (2)	H15A—C15—H15B	109.5
C6—C5—H5A	120.4	O6—C15—H15C	109.5
C4—C5—H5A	120.4	H15A—C15—H15C	109.5
C5—C6—C1	122.0 (2)	H15B—C15—H15C	109.5
C5—C6—H6A	119.0	O7—C16—H16A	109.5
C1—C6—H6A	119.0	O7—C16—H16B	109.5
N2—C7—C8	122.3 (2)	H16A—C16—H16B	109.5
N2—C7—H7A	118.8	O7—C16—H16C	109.5
C8—C7—H7A	118.8	H16A—C16—H16C	109.5
C9—C8—C13	120.9 (2)	H16B—C16—H16C	109.5
			
C1—N1—N2—C7	−174.7 (2)	N2—C7—C8—C9	166.5 (2)
N2—N1—C1—C6	−1.8 (3)	N2—C7—C8—C13	−10.3 (4)
N2—N1—C1—C2	177.9 (2)	C13—C8—C9—C10	1.8 (4)
N1—C1—C2—C3	−176.6 (2)	C7—C8—C9—C10	−175.1 (2)
C6—C1—C2—C3	3.1 (3)	C14—O5—C10—C9	−3.7 (3)
N1—C1—C2—N3	4.2 (4)	C14—O5—C10—C11	177.5 (2)
C6—C1—C2—N3	−176.1 (2)	C8—C9—C10—O5	178.1 (2)
O2—N3—C2—C3	5.1 (4)	C8—C9—C10—C11	−3.1 (4)
O1—N3—C2—C3	−174.5 (2)	C15—O6—C11—C10	−86.0 (3)
O2—N3—C2—C1	−175.7 (3)	C15—O6—C11—C12	97.3 (3)
O1—N3—C2—C1	4.7 (4)	O5—C10—C11—O6	4.6 (3)
C1—C2—C3—C4	−1.7 (4)	C9—C10—C11—O6	−174.2 (2)
N3—C2—C3—C4	177.6 (2)	O5—C10—C11—C12	−178.6 (2)
C2—C3—C4—C5	−1.2 (4)	C9—C10—C11—C12	2.5 (4)
C2—C3—C4—N4	−179.4 (2)	C16—O7—C12—C13	−0.1 (4)
O3—N4—C4—C3	−1.9 (3)	C16—O7—C12—C11	−179.8 (3)
O4—N4—C4—C3	177.5 (3)	O6—C11—C12—O7	−4.0 (3)
O3—N4—C4—C5	179.8 (2)	C10—C11—C12—O7	179.2 (2)
O4—N4—C4—C5	−0.8 (3)	O6—C11—C12—C13	176.3 (2)
C3—C4—C5—C6	2.4 (4)	C10—C11—C12—C13	−0.5 (4)
N4—C4—C5—C6	−179.4 (2)	O7—C12—C13—C8	179.4 (2)
C4—C5—C6—C1	−0.8 (4)	C11—C12—C13—C8	−0.9 (4)
N1—C1—C6—C5	177.8 (2)	C9—C8—C13—C12	0.3 (4)
C2—C1—C6—C5	−1.8 (3)	C7—C8—C13—C12	177.0 (2)
N1—N2—C7—C8	−176.2 (2)		

### Hydrogen-bond geometry (Å, º)

Cg1 is the centroid of the C1–C6 benzene ring.

**Table:**

*D*—H···*A*	*D*—H	H···*A*	*D*···*A*	*D*—H···*A*
N1—H1*N*1···O1	0.82 (2)	2.04 (2)	2.624 (3)	129 (2)
C16—H16*C*···O2^i^	0.96	2.44	3.169 (4)	133
C14—H14*B*···*Cg*1^ii^	0.96	2.89	3.514 (3)	123

Symmetry codes: (i) −*x*+1, *y*+1/2, −*z*+3/2; (ii) −*x*+3/2, −*y*+1, *z*+1/2.
